# Optimal Configuration of Redundant Inertial Sensors for Navigation and FDI Performance

**DOI:** 10.3390/s100706497

**Published:** 2010-07-02

**Authors:** Duk-Sun Shim, Cheol-Kwan Yang

**Affiliations:** School of Electrical and Electronics Engineering, Chung-Ang University, 221 HukSuk-dong, Dongjak ku, Seoul, 156–756, Korea; E-Mail: ckyang92@empal.com

**Keywords:** optimal sensor configuration, redundant inertial sensor, navigation performance, FDI performance, fault detection and isolation, platonic solid, icosahedrons

## Abstract

This paper considers the optimal sensor configuration for inertial navigation systems which have redundant inertial sensors such as gyroscopes and accelerometers. We suggest a method to determine the optimal sensor configuration which considers both the navigation and FDI performance. Monte Carlo simulations are performed to show the performance of the suggested optimal sensor configuration method.

## 1. Introduction

Inertial navigation systems (INS) require at least three accelerometers and three gyroscopes to calculate the navigation information such as the position, velocity and attitude. However, the use of redundant sensors is preferable to ensure their reliability and enhance their navigation accuracy and, thus, the problem of the proper placement of the redundant inertial sensors has been studied since the 1970s. For over four decades reliability has been a subject of interest in various complex systems, such as industrial process systems and power systems, as well as in safety-critical systems such as nuclear power systems and the control of military and space aircraft. Hardware redundancy has been studied from the early stages of the introduction and development of FDI (fault detection and isolation). The various FDI approaches to hardware redundancy include the following methods: the squared-error (SE) [1], generalized likelihood test (GLT) [2], minimax [3], sequential [4], optimal parity test (OPT) [5], multiple parity vectors [6], and double fault isolation method [7]. Wilcox [8] compared the results of some FDI methods. Gai *et al*. [9] analyzed the FDI performance of two redundant sensor configurations. Yang *et al*. [10] suggested the best sensor configuration and accommodation rule for an INS with seven inertial sensors

The optimal configuration problem of redundant inertial sensors was studied in [11], where redundant MEMS-IMU integration with GPS is considered. Recently, finding the optimal sensor configuration for passive source localization [12] or mobile sensor networks [13] has attracted considerable attention. One of the main applications of modern source localization is the surveillance and protection of military, industrial or strongly populated areas [12]. The optimal sensor configuration is necessary to optimize the passive source localization. It is interesting to note that the condition of the optimal sensor configuration for passive position estimation in [12] is the same as the optimal configuration for the redundant inertial sensors in [14].

In the early 1970s, nine inertial sensors were employed in aircraft, with three sensors in each axis, since it was not known how to optimally configure the sensors. One of the earliest references to redundancy in inertial units uses two sets of orthogonal triads skewed against one another [15]. Gilmore *et al.* [1] suggested a symmetric configuration with a dodecahedron and Pejsa [16] suggested optimal configurations for four, five, and six inertial sensors. Much additional research into the optimal configuration of redundant sensors in INS was subsequently performed [1,13,14,16].

Harrison *et al.* [14] suggested the use of figures of merit to evaluate the sensor orientations for navigation performance and FDI capability. With the figure of merit of the navigation performance, the optimal condition for the sensor configuration is obtained and, with the figure of merit of the FDI performance, alternative sensor orientations are evaluated and compared with each other. The condition required to obtain the optimal navigation performance is well-known nowadays, while the condition for the optimal FDI performance is not known yet. Platonic solids (or regular polyhedrons) are known to be the optimal configuration for both the navigation and FDI performance. Thus, when the number of sensors is six or 10, dodecahedrons and icosahedrons are the best sensor configurations for the navigation and FDI performance, respectively. However, when the number of sensors is other than six or 10, such as five, seven or eight, the optimal configuration for both the navigation and FDI performance remains unknown.

In this paper, we focus on hardware redundancy in INS and especially on the optimal configuration and suggest a figure of merit for a sensor configuration considering both the navigation and FDI performance. The proposed figure of merit can be used to compare the alternative sensor configurations and, thus, it is possible to obtain the optimal configuration of the redundant sensors considering both the best navigation and FDI performance. Section 2 discusses the condition of the optimal sensor configuration for the navigation performance and gives some sensor configurations providing the best navigation performance, and Section 3 discusses the FDI performance of the sensor configurations with respect to the number of sensors and the angles between them. Section 4 discusses the main results of this paper and suggests a figure of merit for a sensor configuration considering both the navigation and FDI performance. Section 5 shows some simulation results to confirm the validity of the suggested method and in Section 6 we give our conclusions.

## 2. Optimal Sensor Configuration for Navigation Performance

### 2.1. The Necessary and Sufficient Condition for Best Navigation Performance

Consider an inertial sensor system which uses more than three gyroscopes and three accelerometers. Then, a typical measurement equation for the redundant inertial sensors can be described as follows:

(1) $$m(t)=\mathrm{Hx}(t)+\varepsilon (t),\;\;\;\varepsilon ∼N(0,\rho I_{n})$$

where:

m = [m_1_ m_2_ … m_n_]^T^ ∈ R*^n^*: inertial sensor measurement
H = [h_1_ … h_n_]^T^: *n*×3 measurement matrix with rank(H)=3 and |h_i_|=1, *i*=1, …, *n*
x(t) ∈ R^3^: the triad-solution (acceleration or angular rate)
ε(t)= [ *ε*_1_, *ε*_2_, …, *ε*_n_]^T^ ∈ R^n^ : a measurement noise vector with a normal distribution(white noise).

The triad solution x̂ =[x̂_x_ x̂_y_ x̂_z_]^T^ for x(t) in (1) can be obtained by the least squares method, as follows:

(2) $${\hat{x}}_{\text{LS}}={\left(H^{T}H\right)}^{-1}H^{T}m$$

The navigation solution such as the position, velocity, and attitude, is calculated from x̂(t). Let us define the estimation error of x(t) as e(t) = x(t) − x̂(t). Then, the navigation accuracy of the INS depends on the error covariance:

(3) $$P(t)=E\left[e(t)e{\left(t\right)}^{T}\right]={\left(H^{T}H\right)}^{-1}\rho^{2}.$$

The figure of merit for the navigation performance can be described as follows:

(4) $$J=\mathrm{trace}(P)=E[{\left(x_{x}-{\hat{x}}_{x}\right)}^{2}]+E[{\left(x_{y}-{\hat{x}}_{y}\right)}^{2}]+E[{\left(x_{z}-{\hat{x}}_{z}\right)}^{2}]$$

**Definition 1**: Optimal sensor configuration for navigation performance

For redundant inertial sensor systems, the optimal configuration for the navigation performance is defined as the configuration which minimizes the figure of merit *J* in (4).

**Theorem 1.** Consider the measurement matrix *H* ∈ *R^n^*^×3^ in (1). The necessary and sufficient condition for the sensor configuration with measurement matrix *_H_* to be optimal for the navigation performance is $H^{T}H=\frac{n}{3}I$.

**Proof: (Sufficiency)** Suppose that $H^{T}H=\frac{n}{3}I$ and the eigenvalues of *H^T^H* are *λ*_1_, *λ*_2_, *and λ*_3_. We obtain the inequality $J=\mathrm{trace}(P)=\rho^{2}\mathrm{trace}\{{\left({\mathrm{HH}}^{T}\right)}^{-1}\}=\rho^{2}(\frac{1}{\lambda_{1}}+\frac{1}{\lambda_{2}}+\frac{1}{\lambda_{3}})\geq \frac{3\rho^{2}}{\sqrt[{3}]{\lambda_{1}\lambda_{2}\lambda_{3}}}$ from the relation $\frac{x+y+z}{3}\geq \sqrt[{3}]{xyz}$ and the equality holds when *λ*_1_ = *λ*_2_ = *λ*_3_. Since $\lambda_{1}=\lambda_{2}=\lambda_{3}=\frac{n}{3}$, the figure of merit *J* for the navigation performance is the minimum one and, thus, the measurement matrix *H* is optimal for the navigation performance.

**(Necessity)** Suppose that the sensor configuration with measurement matrix *H* is optimal for the navigation performance, which means that the figure of merit *J* for the navigation performance is the minimum one. Suppose that the eigenvalues of *H^T^H* are *λ*_1_, *λ*_2_, and *λ*_3_. We obtain $J=\mathrm{trace}(P)=\rho^{2}\mathrm{trace}\{{\left({\mathrm{HH}}^{T}\right)}^{-1}\}=\rho^{2}(\frac{1}{\lambda_{1}}+\frac{1}{\lambda_{2}}+\frac{1}{\lambda_{3}})\geq \frac{3\rho^{2}}{\sqrt[{3}]{\lambda_{1}\lambda_{2}\lambda_{3}}}$ from the relation, $\frac{x+y+z}{3}\geq \sqrt[{3}]{\mathrm{xyz}}$, and the equality holds when *λ*_1_ = *λ*_2_ = *λ*_3_. Since *J* is the minimum, the equality holds when *λ*_1_ = *λ*_2_ = *λ*_3_. We know that $\mathrm{trace}({\mathrm{HH}}^{T})=\mathrm{trace}(H^{T}H)=\sum_{i=1}^{n}{\left\|h_{i}\right\|}^{2}=n$ and *trace*(*HH^T^*) = *λ*_1_ + *λ*_2_ + *λ*_3_. From *λ*_1_ = *λ*_2_ = *λ*_3_ and *λ*_1_ + *λ*_2_ + *λ*_3_= *n*, we find that $\lambda_{1}=\lambda_{2}=\lambda_{3}=\frac{n}{3}$. Now, we need to show that $H^{T}H=\frac{n}{3}I$ holds. From the singular value decomposition, the measurement matrix *H* is decomposed into *H* = *UAV^T^* where *U* = [*u*_1_,. . .,*u_n_*], *V* = [*v*_1_, *v*_2_, *v*_3_], $A=\left[\begin{array}{c} \Sigma \\ 0 \end{array}\right]$ and Σ = *diag*{σ_1_, σ_2_, σ_3_}. The vectors *u_i_ and v_i_* are the left and right eigenvalues corresponding to the singular value σ*_i_* of *H*, respectively. Since the matrices *U and V* are unitary and $\sigma_{i}^{2}=\lambda_{i}$, $\Sigma =\mathrm{diag}\{\sqrt{\frac{n}{3}},\sqrt{\frac{n}{3}},\sqrt{\frac{n}{3}}\;\}$, we find that $H^{T}H={\mathrm{VA}}^{T}U^{T}{\mathrm{UAV}}^{T}=\Sigma^{2}=\frac{n}{3}I$.

**Remark 1**: Harrison and Gai [8] used $F_{p}=\sqrt{\left|{\left(H^{T}H\right)}^{-1}\right|}$ as the figure of merit for the navigation performance. However, the figure of merit *F_p_* and *J* in (4) give similar results.

### 2.2. Various Optimal Configurations for Navigation Performance

This section shows that there exist many configurations which provide the best navigation performance. The necessary and sufficient condition for the best navigation performance is $H^{T}H=\frac{n}{3}I$ as stated in Theorem 1. The matrix *H* which satisfies the condition is not unique. Tables 1 through 6 show some configurations which satisfy the condition $H^{T}H=\frac{n}{3}I$ according to the number of sensors.

**Figure 1.**
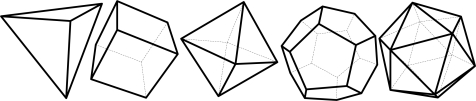
Platonic solids (Regular Polyhedron).

Figure 1 shows the various Platonic solids: tetrahedron, cube, octahedron, dodecahedron, and icosahedrons. The sensor configurations whose input axes are placed perpendicular to the surface of the Platonic solids satisfy the condition, *H^T^H* = (*n*/3)*I*. Thus, Platonic solids provide the optimal navigation performance. The tetrahedron corresponds to the 1^st^ configuration in Table 2, the cube to the configuration in Table 1, the octahedron to the 2nd configuration in Table 2, the dodecahedron to the 1st configuration in Table 4, and the icosahedrons to the 1st and 3rd configurations in Table 6.

There are an infinite number of configurations which satisfy the condition *H^T^H* = (*n*/3)*I* for *n* = 10, as shown in Theorems 2 and 3.

**Theorem 2.** Consider the sensor configuration for *n* = 10 and the measurement matrix *H* which is given as follows:
(5)
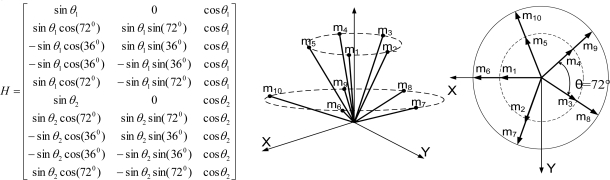
where *θ*_1_ and *θ*_2_ are the angles between the z-axis and the inner and outer cone surfaces, respectively. If ${\text{cos}}^{2}\theta_{1}+{\text{cos}}^{2}\theta_{2}=\frac{2}{3}$ holds, then the measurement matrix *H* satisfies the condition $H^{T}H=\frac{10}{3}I$.

**Proof:** By matrix multiplication, the equation $H^{T}H=\frac{10}{3}I$ can be obtained easily.

**Remark 1:** For Theorem 2, the measurement matrix *H* in (5) becomes the measurement matrix of the icosahedrons when *θ*_1_ = 37.3774^0^, *θ*_2_ = 79.1877^0^, which is the 1^st^ configuration in Table 6.

**Theorem 3**. Consider the sensor configuration for *n* = 10 and the measurement matrix *H* which is given as follows:
(6)
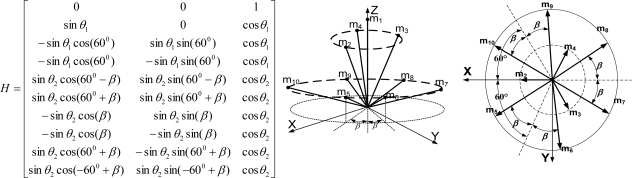
where *θ*_1_ and *θ*_2_ are the angles between the z-axis and the inner and outer cone surfaces, respectively. If ${\text{cos}}^{2}\theta_{1}+2{\text{cos}}^{2}\theta_{2}=\frac{7}{9}$ holds, then the measurement matrix *H* satisfies the condition $H^{T}H=\frac{10}{3}I$ for any *β*.

**Proof**: By matrix multiplication, the equation $H^{T}H=\frac{10}{3}I$ can be obtained easily.

**Remark 2**: For Theorem 3, the measurement matrix *H* in (6) becomes the measurement matrix of the icosahedrons when *θ*_1_ = 41.8103^0^, *θ*_2_ = 70.5288^0^, *β* = 22.2388^0^, which is the 3rd configuration in Table 6.

## 3. Sensor Configuration for FDI Performance

### 3.1. FDI Performance due to the Number of Sensors

When a fault is included in the measurement equation (1), it can be described as follows:

(7) m(t)=Hx(t)+f(t)+ε(t)

where f(t) = [f_1_ f_2_ … f_n_]^T^ ∈ R^n^ is the fault vector.

The parity vector p(t) is calculated from the measurement using the matrix V as follows:

(8) p(t)=Vm(t)=Vf(t)+ Vε(t)

where the matrix V satisfies:

(9) $$\text{VH}=0\;(V\in R^{(n-3)\times n}),\;{\text{VV}}^{T}=I,\;V=[v_{1}\;v_{2}\;\ldots \;v_{n}].$$

The parity vector p(t) is used for fault detection and isolation(FDI) and the matrix V in (9) is used for various algorithms of FDI. The column vector v_i_ has a dimension of (*n*–3)/1. As the number of sensors increases, the dimension of v_i_ increases and thus the FDI performance is enhanced.

**Figure 2.**
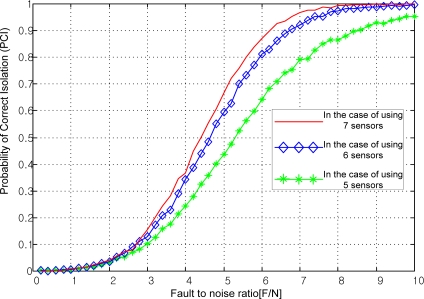
PCI with respect to the number of sensors and fault size.

The FDI performance is related to many parameters such as the existence of a false alarm, miss-detection, correct isolation, and wrong isolation. The probability of correct isolation (PCI) can be used as the main index of the FDI performance. Figure 2 shows that as the fault magnitude to noise ratio increases or as the number of sensors increases, the PCI increases. Cone configurations, *viz.* the first one in Table 3, the second one in Table 4, and the first one in Table 5, are used in the simulation of Figure 2.

**Remark 3**: It is well-known that the navigation performance improves as the number of sensors increases. In other words, the figure of merit for the navigation performance *J* in (4) decreases as the number of sensors increases. The FDI performance shows a similar trend with respect to the number of sensors. That is, as the number of sensors increases, the PCI increases.

### 3.2. Various Optimal Configurations for Navigation Performance

**Figure 3.**
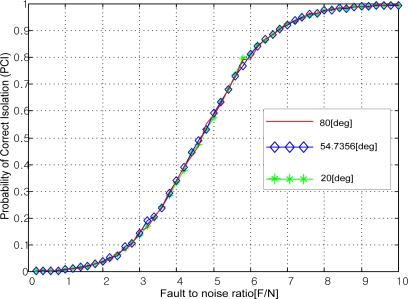
PCI for various cone angles from the center axis with n = 6.

Generally speaking, the wider the orientation vector corresponding to the spread of the inertial sensors, the better the navigation performance. However, this trend does not apply to the FDI performance. For example, consider the cone configuration with six sensors (the second one in Table 4), in which case the cone angle from the center axis is 54.7356°. Figure 3 shows the PCIs for the cone configurations with cone angles of 80° and 20° The simulation result shows that the three PCIs are the same. The V matrices in (8) for the above three cases turn out to be the same, while the measurement matrices are different. Lemma 4 states more general cases of the cone configuration.

**Lemma 4**. Consider a cone configuration H, where the input axes of n sensors are placed on the cone surface evenly and *θ* is the angle between the cone axis and the cone surface. Then, the matrix V satisfying equation (9) is a constant matrix, regardless of the angle, *θ*, and the number of sensors, n.

**Proof.** For the cone configuration, the measurement matrix H can be obtained as follows:

$$H=\left[\begin{array}{ccc} \text{sin}\theta & 0 & \text{cos}\theta \\ \text{sin}\theta \text{cos}(\frac{2\pi }{n}) & \text{sin}\theta \text{sin}(\frac{2\pi }{n}) & \text{cos}\theta \\ \vdots & \vdots & \vdots \\ \text{sin}\theta \text{cos}(k\frac{2\pi }{n}) & \text{sin}\theta \text{sin}(k\frac{2\pi }{n}) & \text{cos}\theta \\ \vdots & \vdots & \vdots \\ \text{sin}\theta \text{cos}((n-2)\frac{2\pi }{n}) & \text{sin}\theta \text{sin}((n-2)\frac{2\pi }{n}) & \text{cos}\theta \\ \text{sin}\theta \text{cos}((n-1)\frac{2\pi }{n}) & \text{sin}\theta \text{sin}((n-1)\frac{2\pi }{n}) & \text{cos}\theta \end{array}\right]$$

The row vectors of the matrix V satisfying VH = 0 forms the null space of H. The range space of H is given as follows: *Range*(*H*) = *span*{*H*_1_,*H*_2_,*H*_3_} where:

$$H_{1}={\left(1\;\;\;\;\;\text{cos}(\frac{2\pi }{n})\;\;\;\;\;\text{cos}(2\frac{2\pi }{n})\;\;\;\;\;\text{cos}(3\frac{2\pi }{n})\cdots \text{cos}((n-1)\frac{2\pi }{n})\right)}^{T},\;\;\;H_{2}={\left(1\;\;\;\;\;\text{sin}(\frac{2\pi }{n})\;\;\;\;\;\text{sin}(2\frac{2\pi }{n})\;\;\;\;\;\text{sin}(3\frac{2\pi }{n})\cdots \text{sin}((n-1)\frac{2\pi }{n})\right)}^{T},\;\;\;H_{3}={\left(1\cdots 1\right)}^{T}.$$

Matrix H depends on *θ*, but *Range*(*H*) does not. Thus, the matrix V does not depend on *θ* and turns out to be a constant matrix, regardless of *θ*.

## 4. Optimal Sensor Configuration for both Navigation and FDI Performance

In this chapter, we suggest a method to provide the optimal sensor configuration from the viewpoint of both the navigation and FDI performance. Chapter II shows that there are many optimal configurations to obtain the best navigation performance for each value of n, the number of sensors. Among the optimal configurations providing the best navigation performance, we need to pick the one that gives the best FDI performance.

Considering both the navigation and FDI performance, we suggest a figure of merit for a sensor configuration H as follows:

(10) $$J_{H}=\underset{i,j(i<j)}{\text{min}}\theta_{\mathrm{ij}}^{H},\;\;\;\;\;\;\;\;\text{subject to}\;H^{T}H=\frac{n}{3}I,\;H\in R^{m\times 3}$$

where $\theta_{\mathrm{ij}}^{H}$ is the angle between the orientation vectors of the i-th and j-th sensors for the sensor configuration, H, and should be calculated so as to be less than a right angle, such that $\theta_{\mathrm{ij}}^{H}=\text{min}\{\theta_{\mathrm{ij}}^{H},\;\;\pi -\theta_{\mathrm{ij}}^{H}\}$. The inner product between *h_i_* and *h_j_* can be used instead of the angle $\theta_{\mathrm{ij}}^{H}$ and another figure of merit for the sensor configuration H can be obtained as follows:

(11) $${\tilde{J}}_{H}=\underset{i,j(i<j)}{\text{max}}|h_{i}h_{j}^{T}|\;\text{subject to}\;H^{T}H=\frac{n}{3}I,\;H\in R^{n\times 3}$$

where H=[h_1_ … h_n_]^T^ and h_i_ is a 3×1 column vector for the configuration H.

Among the configurations providing the best navigation performance, the optimal configuration is the one which makes the angle between the nearest two sensors the largest, which suggests a method to provide the best sensor configuration for both the navigation and FDI performance as follows:

(12) $$H_{\mathrm{optimal}}=\underset{H_{k}}{\text{arg max}}\;\underset{i,j(i<j)}{\text{min}}\;\theta_{\mathrm{ij}}^{H_{k}},\;\text{subject to}\;H_{K}^{T}H_{k}=\frac{n}{3}I,\;H_{k}\in R^{n\times 3},\;k=1,2,3,\;\ldots .$$

The inner product between *h_ki_* and *h_kj_* can be used instead of the angle $\theta_{\mathrm{ij}}^{H_{k}}$ and then *H_optimal_* can be expressed as follows.

(13) $${\tilde{H}}_{\mathrm{optimal}}=\underset{H_{k}}{\text{arg min}}\;\underset{i,j(i<j)}{\text{max}}\;|h_{\mathrm{ki}}h_{\mathrm{kj}}^{T}|\;\text{subject to}\;H_{K}^{T}H_{k}=\frac{n}{3}I,\;H_{k}\in R^{n\times 3},\;k=1,2,3,\;\ldots .$$

Table 7 shows the result of Equations (12) or (13) applied to the configurations in Table 3 through Table 6. The first row in Table 7 is the result of Equations (12) or (13) when five sensors are used. The first configuration in Table 3 gives the maximum (minimum) of the inner product (angles) as 0.5393 (57.3640^0^). Among the three configurations in Table 3, which provide the best navigation performance, the first configuration shows the best FDI performance. Table 7 shows that when 10 sensors are used, configurations 1 and 3 are the best. The reason for this is that configurations 1 and 3 in Table 6 use different sets of sensors from the same icosahedron. Symmetric configurations such as Platonic solids are known to be the best configurations for both the navigation and FDI performance. Table 7 shows that Platonic solids provide the best configuration for both the navigation and FDI performance.

**Remark 4**: Algorithm (13) is preferred to (12), since the calculation of (13) is simpler and only matrix H is used. If we know all of the solutions of H satisfying the equation $H^{T}H=\frac{n}{3}I$, we can obtain the optimal configuration for each value of n. However, we do not know all of the solutions yet, thus we obtain some sets of solutions of $H^{T}H=\frac{n}{3}I$ and then pick the best one among the candidates using (13).

## 5. Simulations

In this chapter, we describe some simulations that were performed to show that the method suggested in (13) works well to obtain the optimal sensor configuration for both the navigation and FDI performance. In Section 5.1, we describe Monte Carlo simulations that were performed to calculate the PCI for the FDI performance, while Section 5.2 describes the simulations conducted using the figure of merit suggested in [8].

### 5.1. Monte Carlo Simulations Using PCI

In this section, we describe the Monte Carlo simulations performed for the configurations in Tables 3 through 6. For each configuration, we assume that a fault occurs and calculate the PCI for the faulty sensor using GLT method [2]. Each PCI is calculated from 3,000 simulation runs and the 3,000 PCIs are averaged to reduce the variation due to noise. The results are given in Tables 8 through 11. For each configuration, the minimum value of the PCI among all of the sensors is underlined. Among the underlined values, the configuration which gives the maximum value is the best one. The results of the best configuration for Tables 8 through 11 are exactly the same as those in Table 7.

### 5.2. Simulation Using the Figure of Merit in [14]

In this section, we calculate the figure of merit for the FDI performance for the configurations in Tables 3 through 6. Harrison and Gai [14] suggested a figure of merit for systematically evaluating alternative sensor configurations. To confirm the results of Section 5.1, the figure of merit in [14] is calculated and the results are shown in Table 12.

A distance measure (14) is used to compare the detectability (and hence the potential FDI performance) inherent in the different configurations of the sensors:

(14) $$J_{j}=v_{j}^{T}{\left(VV^{T}\right)}^{-1}v_{j}$$

which is the distance measure between the statistics for the parity vector with a bias fault and the parity vector without a fault. Since there are n measurements, there is an n-dimensional vector:

(15) $$J^{T}=(J_{1},\;J_{2},\;\cdots ,\;J_{n})$$

The figure of merit is defined as in (16):

(16) $$J_{d1}=\underset{j}{\text{min}}\;\{J_{j}\},\;\;j=1,2,\;\cdots ,\;n$$

where *J_d_*_1_ is thus a measure for the least detectable failure mode and a function of the matrix V. Among the various sensor configurations, the configuration which yields the maximum *J_d_*_1_ is the one which provides the best FDI performance.

The value in the cell of Table 12 is *J_d_*_1_ in (16) for each sensor configuration in Table 3 through Table 6. The results in Table 12 are the same as those in Tables 8 through 11.

## 6. Conclusions

This paper considers the optimal sensor configuration for inertial navigation systems which have redundant inertial sensors. We show that the condition which affords the optimal sensor configuration for the best navigation performance is a necessary and sufficient condition, and enumerate some of the best sensor configurations for navigation performance. We suggest a figure of merit to determine the optimal sensor configuration which considers both the navigation and FDI performance. The main criterion is that among the configurations providing the best navigation performance, the optimal configuration is the one which makes the angle between the nearest two sensors the largest

Monte Carlo simulations are performed to demonstrate the performance of the suggested optimal sensor configuration method. For the FDI performance, the probability of correct isolation is used. To obtain one PCI value in the table, 3,000 Monte Carlo simulation runs are performed and the resulting 3,000 values are averaged. The results of the Monte Carlo simulations were found to be the same as those of the suggested method. The figure of merit (FOM) for the FDI performance suggested in [6] is used to reconfirm the performance of the suggested method, and the FOM results were identical to those of the Monte Carlo simulations.

## Figures and Tables

**Table 1.** Configurations which satisfy $H^{T}H=\frac{n}{3}I$ for *n* = 3.

**Configuration Diagram**	**Measurement Matrix**
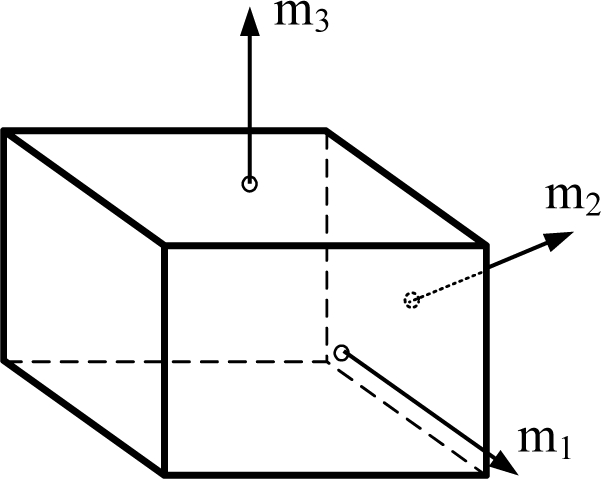	$H=\left[\begin{array}{ccc} 1 & 0 & 0\\ 0 & 1 & 0\\ 0 & 0 & 1 \end{array}\right]$

**Table 2.** Configurations which satisfy $H^{T}H=\frac{n}{3}I$ for *n* = 4.

	**Configuration Diagram**	**Measurement Matrix**
1	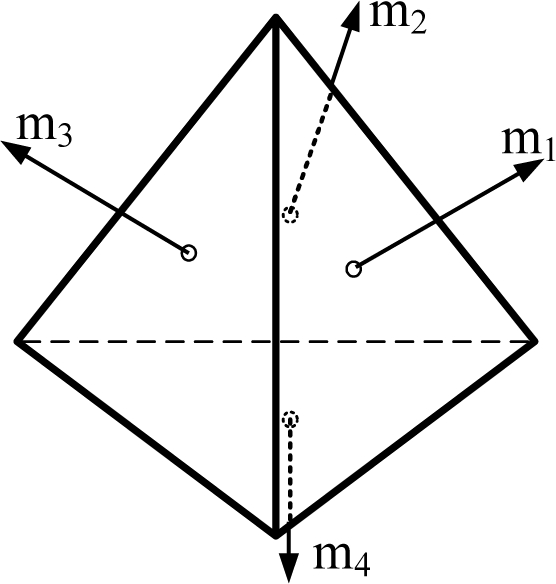	$H=\left[\begin{array}{ccc} \frac{2\sqrt{2}}{3} & 0 & \frac{1}{3}\\ -\frac{\sqrt{2}}{3} & \frac{\sqrt{6}}{3} & \frac{1}{3}\\ -\frac{\sqrt{2}}{3} & -\frac{\sqrt{6}}{3} & \frac{1}{3}\\ 0 & 0 & 1 \end{array}\right]$
2	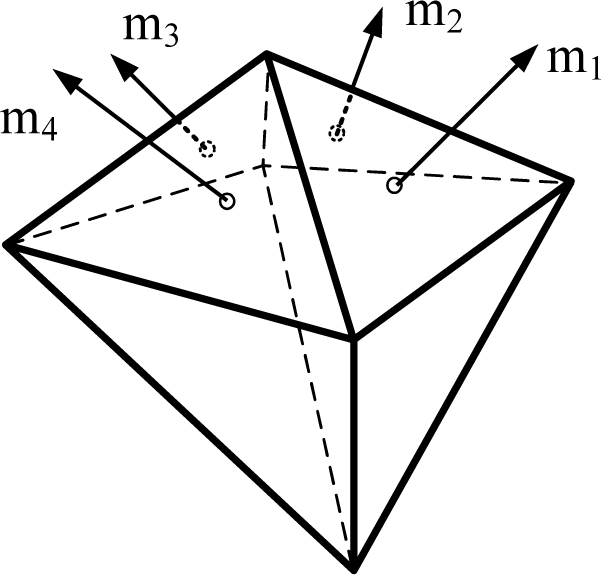	$H=\frac{1}{\sqrt{3}}\left[\begin{array}{ccc} -1 & -1 & 1\\ 1 & -1 & 1\\ 1 & 1 & 1\\ -1 & 1 & 1 \end{array}\right]$

**Table 3.** Configurations which satisfy $H^{T}H=\frac{n}{3}I$ for *n* = 5.

	**Configuration Diagram**	**Measurement Matrix**
1	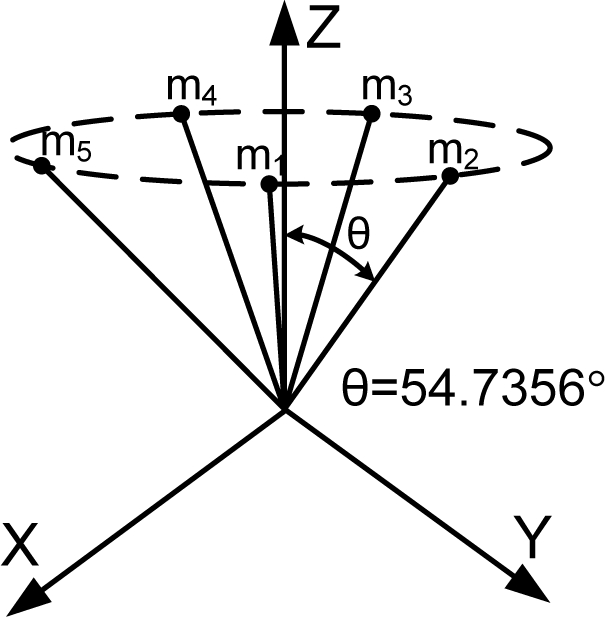	$H=\left[\begin{array}{ccc} 1/\sqrt{3} & 1/\sqrt{3} & 1/\sqrt{3}\\ -0.3707 & 0.7275 & 1/\sqrt{3}\\ -0.8064 & -0.1277 & 1/\sqrt{3}\\ -0.1277 & -0.8064 & 1/\sqrt{3}\\ 0.7275 & -0.3707 & 1/\sqrt{3} \end{array}\right]$
2	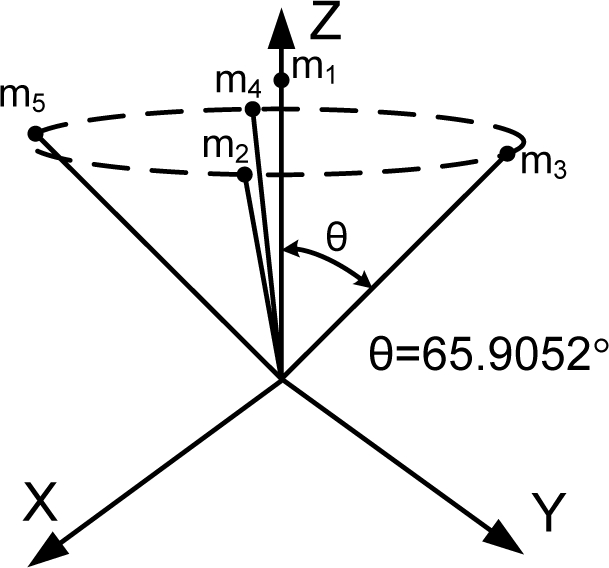	$H=\left[\begin{array}{ccc} 0 & 0 & 1\\ \sqrt{\frac{5}{12}} & \sqrt{\frac{5}{12}} & \frac{1}{\sqrt{6}}\\ -\sqrt{\frac{5}{12}} & \sqrt{\frac{5}{12}} & \frac{1}{\sqrt{6}}\\ -\sqrt{\frac{5}{12}} & -\sqrt{\frac{5}{12}} & \frac{1}{\sqrt{6}}\\ \sqrt{\frac{5}{12}} & -\sqrt{\frac{5}{12}} & \frac{1}{\sqrt{6}} \end{array}\right]$
3	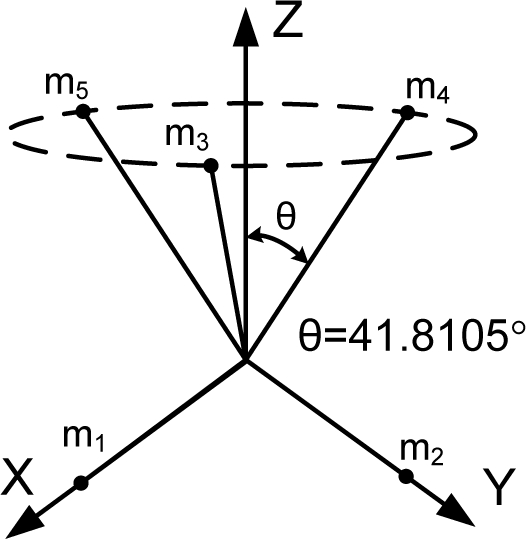	$H=\left[\begin{array}{ccc} 1 & 0 & 0\\ 0 & 1 & 0\\ 0.4714 & 0.4714 & 0.7454\\ -0.6440 & 0.1725 & 0.7454\\ 0.1725 & -0.6440 & 0.7454 \end{array}\right]$

**Table 4.** Configurations which satisfy $H^{T}H=\frac{n}{3}I$ for *n* = 6.

	**Configuration Diagram**	**Measurement Matrix**
1	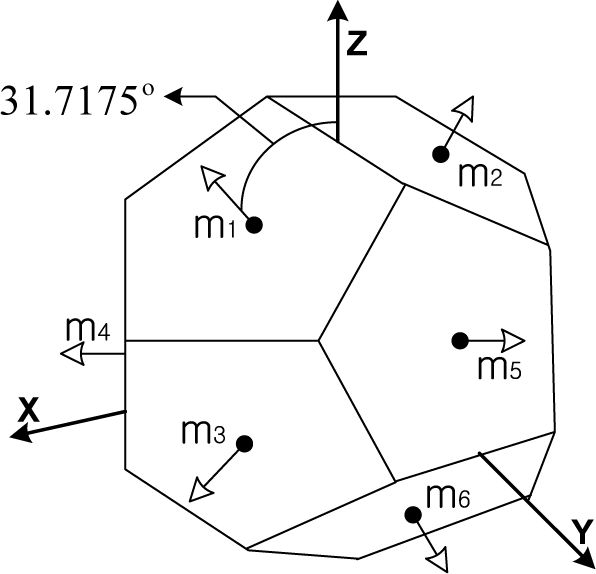	$H=\left[\begin{array}{ccc} 0.5257 & 0 & 0.8507\\ -0.5257 & 0 & 0.8507\\ 0.8507 & 0.5257 & 0\\ 0.8507 & -0.5257 & 0\\ 0 & 0.8507 & 0.5257\\ 0 & 0.8507 & -0.5257 \end{array}\right]$
2	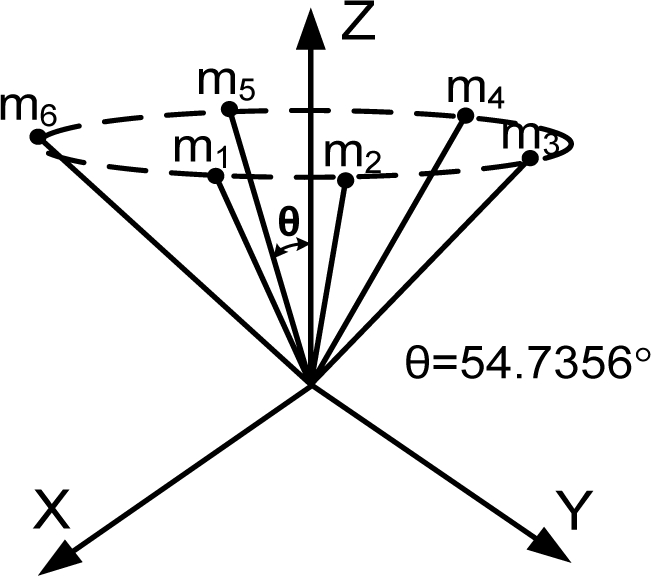	$H=\left[\begin{array}{ccc} 0.8165 & 0 & 0.5744\\ 0.4082 & 0.7071 & 0.5744\\ -0.4082 & 0.7071 & 0.5744\\ -0.8165 & 0 & 0.5744\\ -0.4082 & -0.7071 & 0.5744\\ 0.4082 & -0.7071 & 0.5744 \end{array}\right]$
3	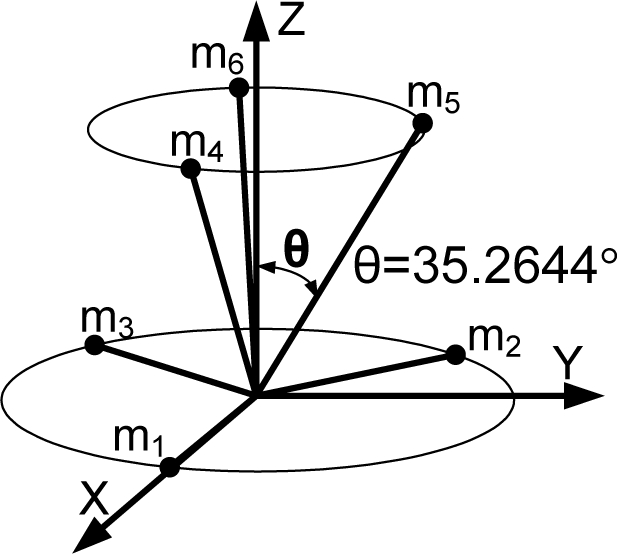	$H=\left[\begin{array}{ccc} 1 & 0 & 0\\ -0.5 & 0.8660 & 0\\ \text{−}0.5 & -0.8660 & 0\\ 0.5774 & 0 & 0.8165\\ -0.2884 & 0.5 & 0.8165\\ -0.2884 & -0.5 & 0.8165 \end{array}\right]$

**Table 5.** Configurations which satisfy $H^{T}H=\frac{n}{3}I$ for *n* = 7.

	**Configuration Diagram**	**Measurement Matrix**
1	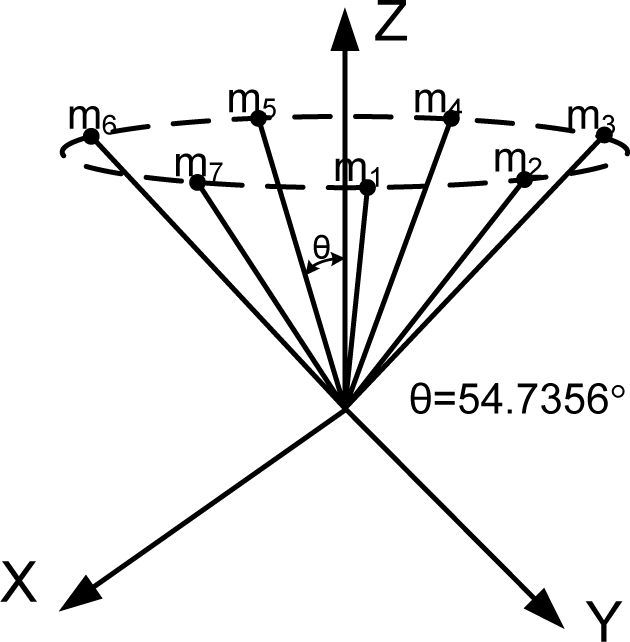	$H=\left[\begin{array}{ccc} 0.8165 & 0 & 0.5774\\ 0.5091 & 0.6384 & 0.5774\\ -0.1817 & 0.7960 & 0.5774\\ -0.7356 & 0.3543 & 0.5774\\ -0.7356 & -0.3543 & 0.5774\\ -0.1817 & -0.7960 & 0.5774\\ 0.5091 & -0.6384 & 0.5774 \end{array}\right]$
2	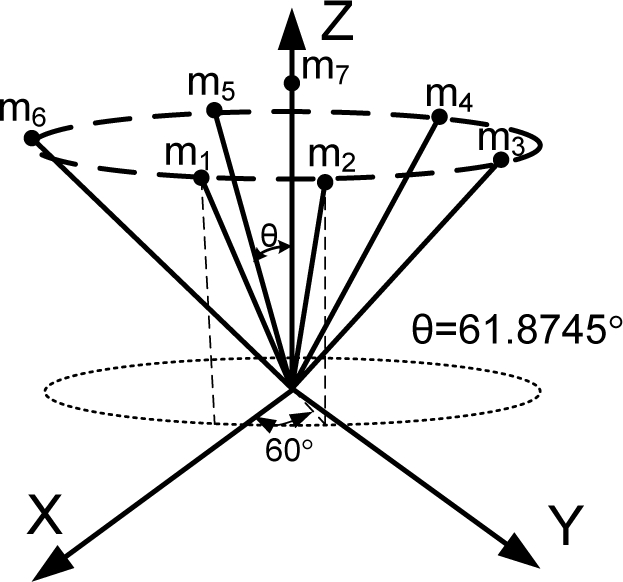	$H=\left[\begin{array}{ccc} 0.8819 & 0 & 0.4714\\ 0.4410 & 0.7638 & 0.4714\\ -0.4410 & 0.7638 & 0.4714\\ -0.8819 & 0 & 0.4714\\ -0.4410 & -0.7638 & 0.4714\\ 0.4410 & -0.7638 & 0.4714\\ 0 & 0 & 1 \end{array}\right]$
3	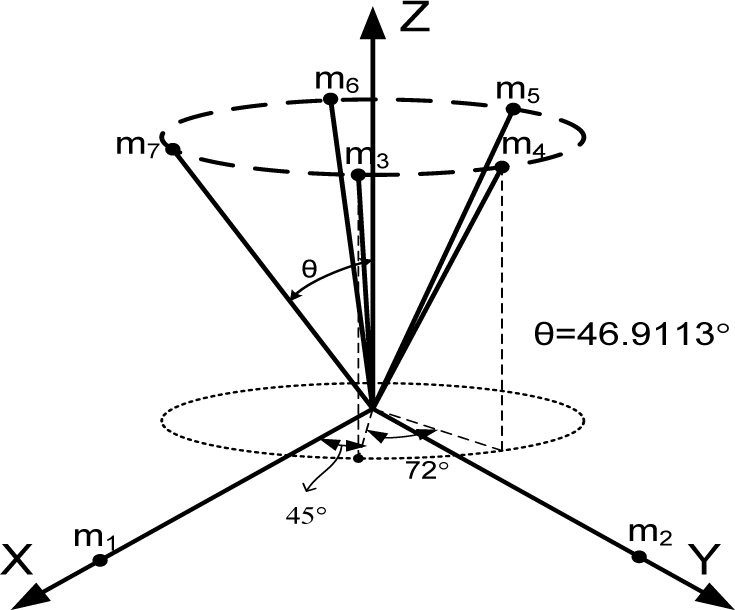	$H=\left[\begin{array}{ccc} 1 & 0 & 0\\ 0 & 1 & 0\\ 0.5164 & 0.5164 & 0.6831\\ -0.3315 & 0.6507 & 0.6831\\ -0.7213 & -0.1142 & 0.6831\\ -0.1142 & -0.7213 & 0.6831\\ 0.6507 & -0.3315 & 0.6831 \end{array}\right]$
4	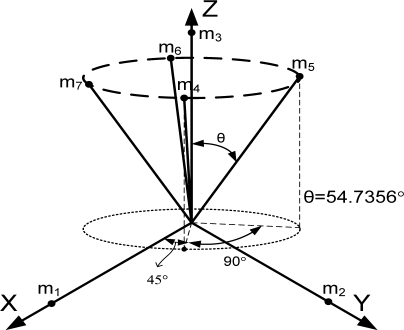	$H=\left[\begin{array}{ccc} 1 & 0 & 0\\ 0 & 1 & 0\\ 0 & 0 & 1\\ 0.5774 & 0.5774 & 0.5774\\ -0.5774 & 0.5774 & 0.5774\\ -0.5774 & -0.5774 & 0.5774\\ 0.5774 & -0.5774 & 0.5774 \end{array}\right]$

**Table 6.** Configurations which satisfy $H^{T}H=\frac{n}{3}I$ for *n* = 10.

	**Configuration Diagram**	**Measurement Matrix**
1	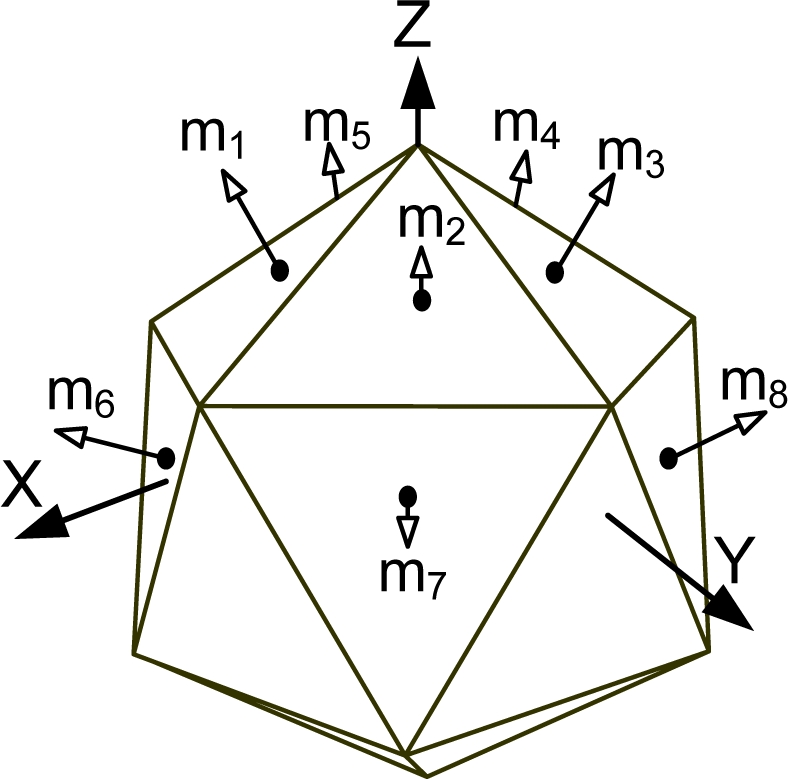	$H=\left[\begin{array}{ccc} 0.7071 & 0 & 0.7071\\ 0.2185 & 0.6725 & 0.7071\\ -0.5721 & 0.4156 & 0.7071\\ -0.5721 & -0.4156 & 0.7071\\ 0.2185 & -0.6725 & 0.7071\\ 0.9280 & 0 & 0.3727\\ 0.2868 & 0.8825 & 0.3727\\ -0.7507 & 0.5454 & 0.3727\\ -0.7507 & -0.5454 & 0.3727\\ 0.2868 & -0.8825 & 0.3727 \end{array}\right]$
2	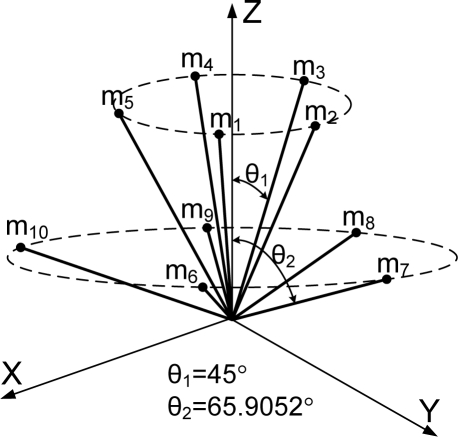	$H=\left[\begin{array}{ccc} 0.7071 & 0 & 0.7071\\ 0.2185 & 0.6725 & 0.7071\\ -0.5721 & 0.4156 & 0.7071\\ -0.5721 & -0.4156 & 0.7071\\ 0.2185 & -0.6725 & 0.7071\\ 0.9129 & 0 & 0.4082\\ 0.2821 & 0.8682 & 0.4082\\ -0.7385 & 0.5366 & 0.4082\\ -0.7385 & -0.5366 & 0.4082\\ 0.2821 & -0.8682 & 0.4082 \end{array}\right]$
3	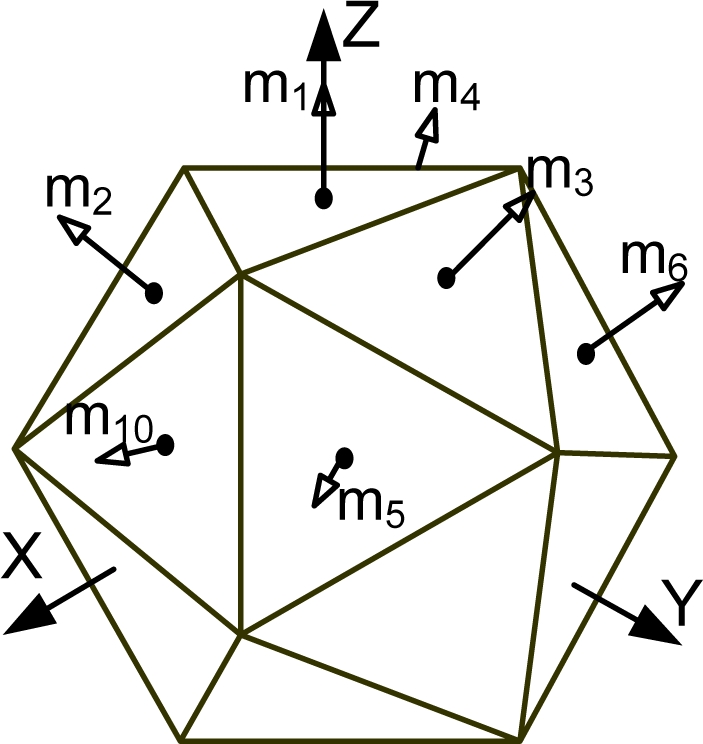	$H=\left[\begin{array}{ccc} 0 & 0 & 1\\ 0.6667 & 0 & 0.7454\\ -1/3 & 0.5774 & 0.7454\\ -1/3 & -0.5774 & 0.7454\\ 0.7454 & 0.5773 & 1/3\\ 0.1273 & 0.9342 & 1/3\\ -0.8727 & 0.3568 & 1/3\\ -0.8727 & -0.3568 & 1/3\\ 0.1273 & -0.9342 & 1/3\\ 0.7454 & -0.5773 & 1/3 \end{array}\right]$
4	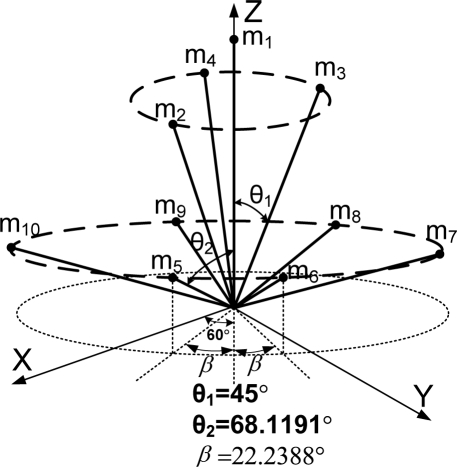	$H=\left[\begin{array}{ccc} 0 & 0 & 1\\ 0.7071 & 0 & 0.7071\\ -0.3536 & 0.6124 & 0.7071\\ -0.3536 & -0.6124 & 0.7071\\ 0.7336 & 0.5683 & 0.3727\\ 0.1253 & 0.9195 & 0.3727\\ -0.8589 & 0.3512 & 0.3727\\ -0.8589 & -0.3512 & 0.3727\\ 0.1253 & -0.9195 & 0.3727\\ 0.7336 & -0.5683 & 0.3727 \end{array}\right]$

**Table 7.** Best configuration for both navigation and FDI performance in Tables 3 through 6.

Sensor Configurations	1	2	3	4	Best Configuration
Number of Sensors					

5	0.5393(57.3640°)	0.6667(48.1871°)	0.6640(48.3943°)	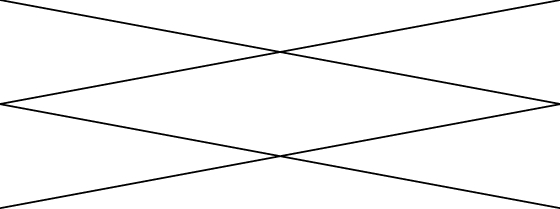	1
6	0.4472(63.4358°)	0.6667(48.1871°)	0.5774(54.7321°)		1
7	0.7491(41.4875°)	0.6111(52.3309°)	0.7213(43.8381°)	0.5774(54.7321°)	4
10	0.7454(41.8065°)	0.9342(20.9007°)	0.7454(41.8065°)	0.7823(38.5284°)	1, 3

**Table 8.** PCI for each faulty sensor with *n* = 5.

Faulty Sensor	1st	2nd	3rd	4th	5th	Best Configuration
Sensor Configurations						

1	0.2443	0.2442	0.2442	0.2445	**0.2441**	best
2	0.3084	0.2050	0.1517	*0.1263*	0.1800	
3	0.1994	*0.1991*	0.2842	0.2219	0.2213	

**Table 9.** PCI for each faulty sensor with *n* = 6.

Faulty Sensor	1st	2nd	3rd	4th	5th	6th	Best Configuration
Sensor Configurations							

1	0.3528	0.3527	0.3524	0.3527	0.3527	**0.3524**	best
2	0.2117	0.2118	*0.2117*	0.2123	0.2123	0.2120	
3	0.3492	0.3491	0.3490	0.3485	*0.3480*	0.3486	

**Table 10.** PCI for each faulty sensor with *n* = 7.

Faulty Sensor	1st	2nd	3rd	4 th	5 th	6 th	7 th	Best Configuration
Sensor Configurations								

1	0.3793	0.3798	0.3796	0.3798	0.3800	0.3793	*0.3791*	
2	0.3852	0.3845	*0.3843*	0.3846	0.3846	0.3848	0.3869	
3	0.3825	0.3819	0.3838	0.3830	0.3812	*0.3811*	0.3828	
4	**0.3847**	0.3849	0.3850	0.3858	0.3858	0.3855	0.3854	best

**Table 11.** PCI for each faulty sensor with *n* = 10.

Faulty Sensor	1st	2nd	3rd	4 th	5 th	6 th	7 th	8 th	9 th	10 th	Best Configuration
Sensor Configurations											

1	0.4101	0.4102	0.4103	0.4106	0.4103	0.4104	0.4103	**0.4101**	0.4105	0.4104	best
2	0.4095	*0.4091*	0.4103	0.4096	0.4099	0.4102	0.4107	0.4098	0.4109	0.4100	
3	**0.4101**	0.4109	0.4103	0.4105	0.4103	0.4102	0.4106	0.4097	0.4102	0.4107	best
4	0.4106	0.4104	0.4104	0.4103	0.4106	0.4105	*0.4100*	0.4103	0.4110	0.4102	

**Table 12.** FDI figures of merit for configurations in Tables 3 through 6.

Sensor Configurations	1	2	3	4	Best Configuration
Number of Sensors					
5	1.5277	1.0000	1.0718	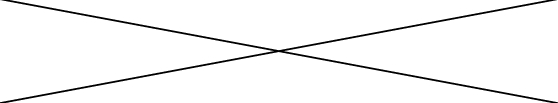	1
6	5.0000	2.2498	3.0000	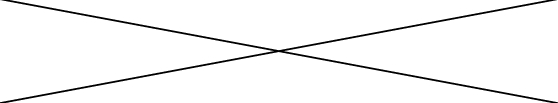	1
7	3.1687	4.7606	3.4165	5.3343	4
10	9.8000	6.2388	9.8000	8.8969	1,3
